# Real-world Implementation of an eHealth System Based on Artificial Intelligence Designed to Predict and Reduce Emergency Department Visits by Older Adults: Pragmatic Trial

**DOI:** 10.2196/40387

**Published:** 2022-09-08

**Authors:** Joël Belmin, Patrick Villani, Mathias Gay, Stéphane Fabries, Charlotte Havreng-Théry, Stéphanie Malvoisin, Fabrice Denis, Jacques-Henri Veyron

**Affiliations:** 1 Hôpital Charles Foix Assistance Publique-Hôpitaux de Paris Ivry-sur-Seine France; 2 Laboratoire Informatique Médicale et Ingénierie des Connaissances en eSanté (UMRS 1142) Institut National de la Santé et de la Recherche Médicale and Sorbonne Université Paris France; 3 Unité de Médecine Interne, Gériatrie et Thérapeutique Assistance Publique-Hôpitaux de Marseille Marseille France; 4 Etablissement Français du Sang, Anthropologie bio-culturelle, Droit, Ethique et Santé Centre National de la Recherche Scientifique Université Aix-Marseille Marseille France; 5 Communauté professionnelle de santé Itinéraire Santé Marseille France; 6 Intervenants Libéraux et Hospitaliers Unis pour le Patient Marseille France; 7 Presage Paris France; 8 Centre hospitalo-universitaire La Réunion Saint-Pierre French Southern Territories; 9 Institut Inter-Régional de Cancérologie Jean Bernard Le Mans France

**Keywords:** emergency department visits, home care aides, community-dwelling older adults, smartphone, mobile phone, predictive tool, health intervention, machine learning, predict, risk, algorithm, model, user experience, alert, monitoring

## Abstract

**Background:**

Frail older people use emergency services extensively, and digital systems that monitor health remotely could be useful in reducing these visits by earlier detection of worsening health conditions.

**Objective:**

We aimed to implement a system that produces alerts when the machine learning algorithm identifies a short-term risk for an emergency department (ED) visit and examine health interventions delivered after these alerts and users’ experience. This study highlights the feasibility of the general system and its performance in reducing ED visits. It also evaluates the accuracy of alerts’ prediction.

**Methods:**

An uncontrolled multicenter trial was conducted in community-dwelling older adults receiving assistance from home aides (HAs). We implemented an eHealth system that produces an alert for a high risk of ED visits. After each home visit, the HAs completed a questionnaire on participants’ functional status, using a smartphone app, and the information was processed in real time by a previously developed machine learning algorithm that identifies patients at risk of an ED visit within 14 days. In case of risk, the eHealth system alerted a coordinating nurse who could then inform the family carer and the patient’s nurses or general practitioner. The primary outcomes were the rate of ED visits and the number of deaths after alert-triggered health interventions (ATHIs) and users’ experience with the eHealth system; the secondary outcome was the accuracy of the eHealth system in predicting ED visits.

**Results:**

We included 206 patients (mean age 85, SD 8 years; 161/206, 78% women) who received aid from 109 HAs, and the mean follow-up period was 10 months. The HAs monitored 2656 visits, which resulted in 405 alerts. Two ED visits were recorded following 131 alerts with an ATHI (2/131, 1.5%), whereas 36 ED visits were recorded following 274 alerts that did not result in an ATHI (36/274, 13.4%), corresponding to an odds ratio of 0.10 (95% IC 0.02-0.43; *P*<.001). Five patients died during the study. All had alerts, 4 did not have an ATHI and were hospitalized, and 1 had an ATHI (*P=*.04). In terms of overall usability, the digital system was easy to use for 90% (98/109) of HAs, and response time was acceptable for 89% (98/109) of them.

**Conclusions:**

The eHealth system has been successfully implemented, was appreciated by users, and produced relevant alerts. ATHIs were associated with a lower rate of ED visits, suggesting that the eHealth system might be effective in lowering the number of ED visits in this population.

**Trial Registration:**

clinicaltrials.gov NCT05221697; https://clinicaltrials.gov/ct2/show/NCT05221697.

## Introduction

The aging human population is increasing worldwide, and their health is characterized by high prevalence of chronic diseases and multimorbidity and a high vulnerability to acute diseases [1,2]. A large proportion of older adults go through emergency department (ED) visits and unplanned hospitalizations, and this proportion increases with advancing age and frailty [3]. In the United States, almost one of every three US emergency medical services responses involves an older adult [4]. In 80% of cases, an older adult’s ED visit is followed by an unscheduled hospitalization, and therefore, has a high medical and economic cost [4]. ED visits and hospitalizations can have a negative impact on the health status of frail older patients by decreasing their functional capacities, which may persist for a long time thereafter [5,6]. Since a large proportion of ED visits are avoidable (range 8%-62%) [7-10], strategies to identify high-risk patients and enable them to be treated in outpatient care settings might help improve the appropriate use of ED visits and control health expenditures [11].

Patient (or family)-reported outcome measure (PROM) systems benefit patients with chronic diseases by improving quality of life, reducing mortality, reducing ED visits, and hospitalizations [12-14]. In 2019, we conducted an observational cohort study, enrolling 301 older individuals who received regular visits by home aides (HAs); we developed a machine learning algorithm to predict the risk of emergency visits, with a prediction window of 7-14 days and a predictive performance (ie, the area under the receiver operating characteristic curve) of 0.70 after 7 days and 0.67 after 14 days [15]. This algorithm opens the possibility of mobilizing health professionals to intervene early in an acute illness or in the decompensation of a chronic illness before they lead to an ED visit and unplanned hospitalization. This represents a significant advance over existing scores with a predictive window of 6-24 months [16-18], which can lead to preventive actions that are temporally distant from events that lead to emergencies.

Today, attending physicians or nurses no longer have time and opportunity to regularly visit older people at home. HAs are key professionals in maintaining older adults at home. They have regular contact with them and can provide important information for decompensation prevention. The idea of this system is to optimize medical interventions when they are really necessary and to find alternatives via health recommendations or other interventions that do not require attending physicians when their presence is dispensable. It allows us to value HAs’ job, and it is based on their proximity with older adults to optimize the care pathway and avoid ED visits. Very few studies in the literature have analyzed the effectiveness of community-based interventions to prevent avoidable emergency hospitalizations of older individuals [16,17]. Recently, Nord et al [18] obtained a 17% decrease in hospitalization rate of older adults in primary care settings, by providing a nurse visit based on comprehensive geriatric assessment among older adults considered at risk for an ED visit by a 12-month predictive tool.

We have conducted a real-world pragmatic trial that included older adults receiving assistance from HAs. We aimed to implement a system that produces alerts when the machine learning algorithm identifies a short-term risk for an ED visit and examine health interventions required after these alerts and users’ experience. This study highlights the feasibility of the general system, the levers for compliance improvement, and its optimal effectiveness in reducing emergency hospitalizations among older people living at home.

## Methods

### Study Design and Recruitment

This multicenter uncontrolled pragmatic trial (NCT05221697) was conducted with 3 home aid facilities participating in the study, located in 3 French cities: Marseille, Versailles, and Dinan. To be eligible, participants should be aged ≥75 years and living at home, receive the help of a social worker from these facilities, have seen their general practitioner within the last 12 months and had a mild or moderate level of dependency according to French national dependency tool, the AGGIR scale [19]. Written consent was obtained before inclusion in the study. Screening and enrollment started on July 1, 2020, and data were collected from September 1, 2020, to August 31, 2021. Participants’ family caregivers and general practitioners received information about the participation of their proxy or patient in the study, as well as the patient’s nurse if the patient received nursing care at home. Participants’ demographics, housing, family situation, dependency level, hospitalization (dates), and death information were collected by the managers of the home aid facilities.

### Intervention

**Figure 1:**
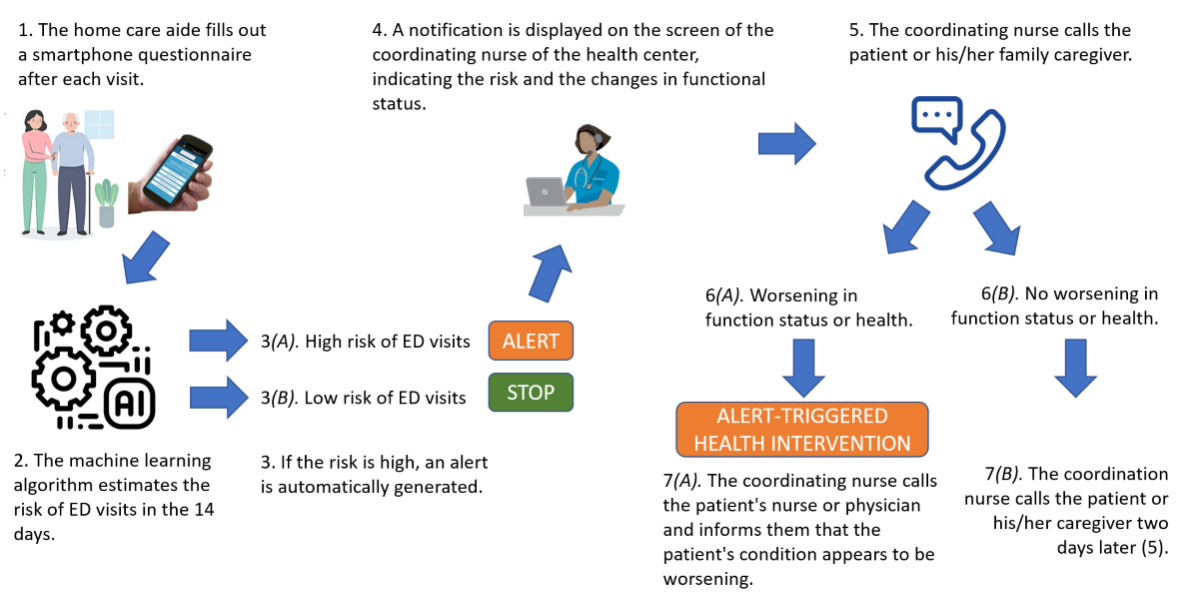
The application of the intervention protocol for alert management. ED: emergency department.

The intervention is summarized in Figure 1. HAs of these facilities were equipped with a smartphone app and were provided with a user manual, defining the app functioning. HAs were asked to complete a simple questionnaire after each home visit, via the smartphone app (Multimedia Appendix 1), which included a user manual, defining the different items in the questionnaire and the answer options.

This questionnaire focused on functional and clinical autonomy (ie, activities of daily life), possible medical symptoms (eg, fatigue, falls, and pain), changes in behavior (eg, recognition and aggressiveness), and communication with the HA or their surroundings. This questionnaire is composed of very simple and easy-to-understand questions, giving a global view of the person’s condition. For each of the 23 questions, a yes/no answer was requested. Data recorded by HAs were sent in real time to a secure server to be analyzed by our machine learning algorithm, which predicted the risk level and displayed it on a web-based secure medical device called PRESAGE CARE, which is CE marked. A simplified diagram of the processing system and the description of data processing system can be found in Multimedia Appendix 2. Particularly, when the algorithm predicted a high-risk level, an alert was displayed in the form of a notification on the screen to the coordinating nurse of the health care network center of the district (Dispositif d’Appui à la Coordination of the Agence Régionale de Santé). This risk notification was accompanied by information about recent changes in the patients’ functional status, identified from the HAs’ records, to assist the coordinating nurse in interacting with family caregiver and other health professionals.

In the event of an alert, the coordinating nurse called the family caregiver to inquire about recent changes in the patient’s health condition and for doubt removal and could then decide to ask for a health intervention according to a health intervention model developed before the start of the study. In brief, this alert-triggered health intervention (ATHI) consisted of calling the patient’s nurse (if the patient had regular home visits of a nurse) or the patient’s general practitioner and informing them of a worsening of the patient’s functional status and a potential risk of an ED visit in the next few days according to the eHealth system algorithm. The ATHI was performed with the natural resources of the health system and not with the physicians or nurses employed in the study. No specific instruction or protocol was given to these health professionals, and they were free to make their own decisions. This model of ATHI had been presented and approved by the Agences Régionales de Santé of the regions involved in our study (Figure 1).

### Outcomes

The primary outcomes were the rate of ED visits and the number of deaths recorded by the coordinating nurses, as well as the users’ experience with HAs and coordinating nurses recorded by the questionnaires. The eHealth system’s organizational outcomes in accordance with the guidelines for the evaluation of eHealth systems of the Haute Autorité de Santé, the French national health agency [20], were as follows: the number of monitored visits, defined as home visits with HAs observations; the alert rate, defined as the ratio of the number of alerts to the number of monitored visits; the intervention rate, defined as the proportion of alerts that led to health interventions; the response time, defined as the length of time from the day of the alert to the day of the intervention; and the nature of the health interventions. To analyze HAs adherence to the eHealth system application, we calculated the rate of HA-monitored visits as the ratio of the number of visits that resulted in observation records to the total number of HA visits recorded by the home care facility managers. Users’ experience with this system was assessed according to Basch et al [21], using 2 self-administered anonymous questionnaires, one for the HAs and the facility managers, and one for the coordinating nurses who participated.

The secondary outcomes were to confirm the predictive capacity of the AI in real-world conditions. The diagnosis accuracy of the eHealth system alerts to predict ED visits was assessed by sensibility, specificity, positive and negative predictive values, and likelihood ratios. Accuracy analysis and reporting was conducted according to Standards for Reporting Diagnostics Accuracy Studies guidelines. Occurrences and dates of ED visits and hospitalizations were recorded by the HAs in each visit and by the home aid facility manager.

### Statistical Analysis

Continuous variables were described by means and SD, or medians and IQRs, if not normally distributed; categorical variables were described by relative frequencies. The 2-tailed *t* test or Wilcoxon test was used to compare quantitative variables and the chi-square or Fisher exact test was used for qualitative variables. Sensitivity, specificity, positive and negative predictive value, and positive and negative likelihood ratios were estimated for each one of the alert visits (by an index test) in relation to an ED visit, that was considered the target condition of the reference standard. *P* values <.05 were considered statistically significant. Statistics were conducted using Stata software (version 16; StataCorp LLC).

### Ethics Approval

The research protocol was approved by the national French ethics committee for biomedical research, the Comité de Protection des Personnes, and the French Agency for the Safety of Health Products (2021-A02131-40–CPP 1-21-072 / 21.02093.000019).

Participants and the HAs and the home aid facilities’ managers were informed about the nature and purpose of the study and provided their written consent accordingly.

## Results

### Participants and Home Aid Professionals Involved

Among beneficiaries of the home aid facilities, 293 individuals were eligible, and 206/293 (71%) agreed to participate in the study and were included. Their mean age was 85 (SD 8) years, 161 of 206 (78%) participants were women, and 94 (45%) had a dependency level of GIR 3 or 4 (ie, moderate dependence level; Table 1).

**Figure 2:**
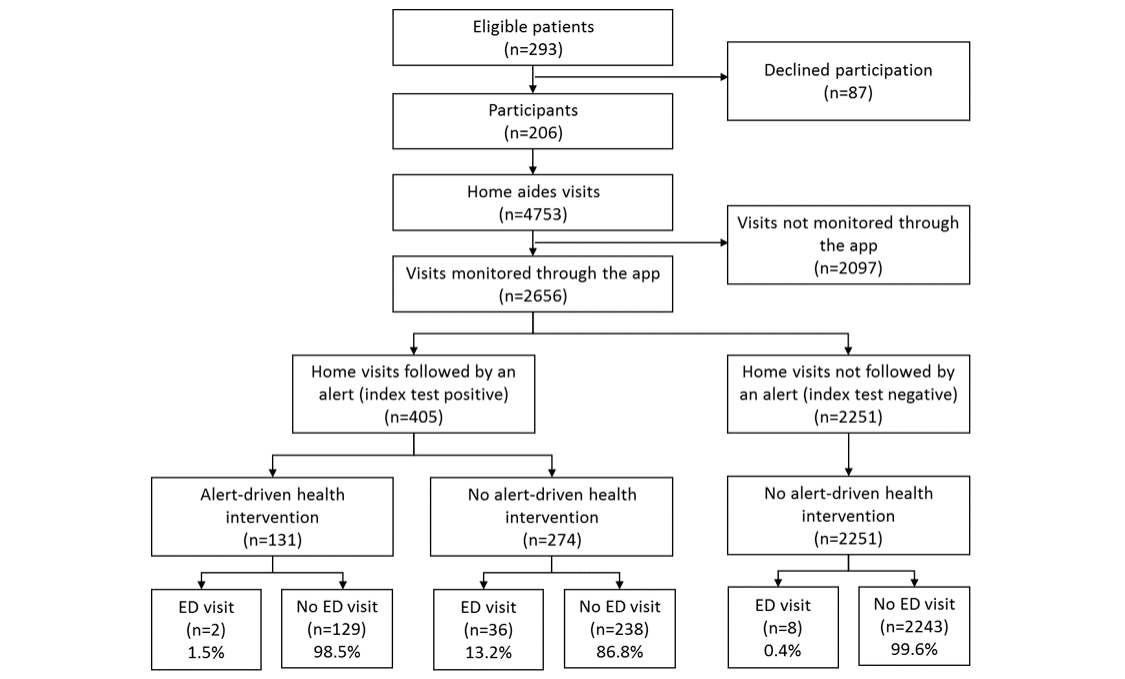
The flowchart of alerts (index test), health interventions, and emergency department (ED) visits (reference standard).

The mean follow-up period was 10 months with no patient loss during the trial. In total, 10 care managers of the home aid facilities (9 nurses and 1 pharmacist) and 109 HAs were involved in the study. From the 4753 home visits, 2656 (56%) were monitored by the app and provided inputs for the eHealth system (Figure 2).

**Table 1:** Participants’ characteristics, activity, and the eHealth system; staff involved in their functioning; and alert-triggered health interventions.

Participants characteristics			Center 1 (n=67)		Center 2 (n=16)		Center 3 (n=123)		Total (N=206)
Age (years), mean (SD)			86 (4)		88 (6)		86 (5)		86 (5)
Gender (women), n (%)			57 (86)		13 (80)		106 (86)		176 (85)
Mild dependency (GIR 5 or 6), n (%)			10 (15)		3 (19)		4 (3)		17 (8)
Moderate dependency (GIR 3 or 4), n (%)			23 (34)		8 (50)		63 (51)		94 (46)
Severe dependency (GIR 1 or 2), n (%)			0 (0)		1 (6)		13 (11)		14 (7)
Unknown dependency level, n (%)			34 (51)		4 (25)		43 (35)		81 (39)
**eHealth system activity**									
	Home aides, n (%)	46 (42)		11 (10)		52 (48)		109 (100)	
	Care managers, n (%)	6 (60)		2 (20)		2 (20)		10 (100)	
	Visits monitored through the app, n (%)	1130 (43)		324 (12)		1202 (45)		2656 (100)	
	Compliance rate, %	56.2		67·5		52.8		56.0	
	Alerts, n (%)	188 (46)		47 (12)		170 (42)		405 (100)	
	Alert rate per monitored visits, %	16.6		14.5		14.1		16.9	
**Alert-triggered health interventions**									
	Interventions, n (%)	45 (34)		46 (35)		40 (31)		131 (100)	
	Intervention rate per alerts, %	23.9		97.9		23.5		32.3	

### Emergency Visits During the Study

Of 206 participants, 29 (14%) visited EDs during the study. Of these, 11 made 2 or more visits (up to 6 visits), and the total number of ED visits was 46; a total of 32 ED visits (for 19 people) were followed by hospitalization.

Hospitalization by direct admission without passing through an ED visit was recorded in 5 participants (Figure 2).

### Health Interventions and ED Visits Occurring After an Alert Display

As a result of the 405 alerts generated by the eHealth system, 131 ATHI by health professionals were performed: 96 (73%) by nurses and 35 (27%) by physicians. After the 131 ATHI, only 2 ED visits (2/131, 1.5%) were recorded, whereas after the 272 alerts that did not result in a health intervention, we recorded 32 ED visits (13.2%), corresponding to an odds ratio of 0.10 (95% IC 0.02-0.43; *P*<.001; Table 2). These health interventions were performed by the patient’s nurse or general practitioner.

Five patients died during the study. All had alerts, 4 did not have ATHI and were hospitalized, and 1 had an ATHI (*P=*.04; Table 3).

**Table 2:** Emergency department (ED) visits that occurred within 14 days of alerts generated by the eHealth system, according to the implementation of a health intervention triggered by the alerts.

Characteristics	ED visits (n=38)	No ED visits (n=367)	Odds ratio (95% CI)
No alert-triggered health intervention, n (%)	36 (13.1)	238 (86.9)	Reference
Alert-triggered health intervention, n (%)	2 (1.5)	129 (98.5)	0.10 (0.02-0.43)^a^

^a^*P*<.001; *P*<.05 is considered statistically significant.

**Table 3:** Association between intervention and death.

		Alert-triggered health interventions				*P* value
		No (n=74)		Yes (n=132)		
**Death, n (%)**						.04
	No (n=201)	70 (94.6)	131 (99.2)			
	Yes (n=5)	4 (5.4)	1 (0.8)			

### Reports of Users’ Experience

**Figure 3:**
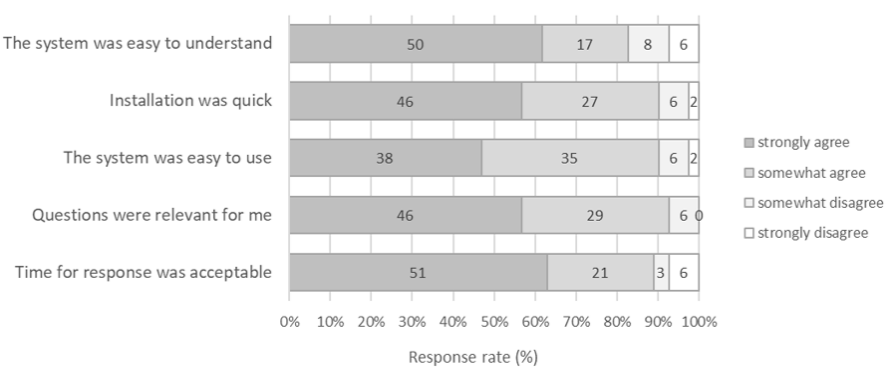
The satisfaction of home aides about the eHealth system.

Users’ experience surveys were completed by 81 of 109 (72%) HAs involved in the study and 8 of 10 (80%) coordinating nurses. In terms of understanding the approach, 83% (90/109) of HAs reported that the screening questions were easy to understand. In terms of overall usability, the digital system was easy to use for 90% (73/81) of HAs, and the response time was acceptable for 89% (72/81) of them (Figure 3).

**Figure 4:**
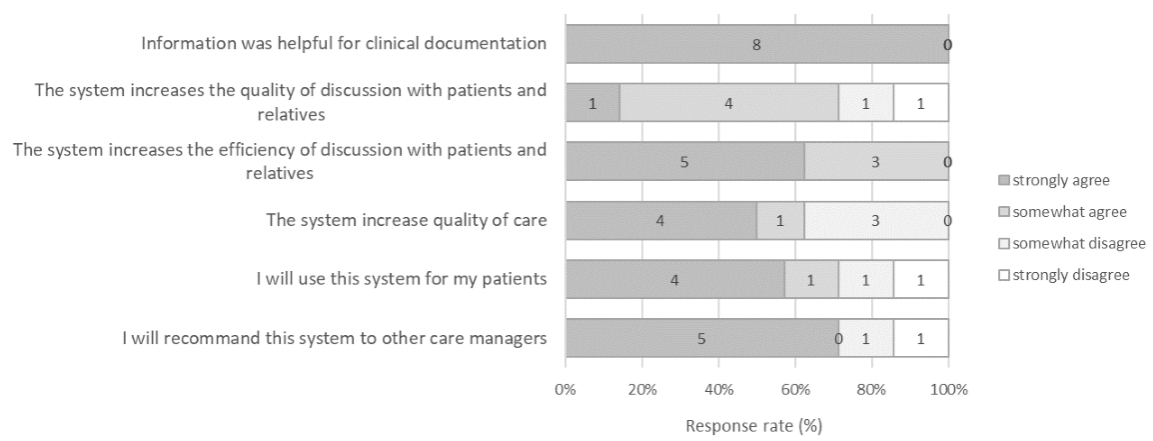
User’s experience with 8 coordinating nurses who received the alerts and completed the questionnaire.

The eHealth system was also well perceived by the coordinating nurses (Figure 4). Most of them found the app questions relevant; they believed the eHealth system had clinical utility and might improve interactions with patients and their family caregiver, and they mentioned that they would like to use it in the future and would recommend it to other facilities.

### The Alerts and Their Prediction of Emergency Department Visit

During the study, 405 alerts (between 22 and 49 per month) were displayed, corresponding to 15.2% (405/2656) of the monitored visits. Of the 46 ED visits, 8 (17%) were not preceded by alerts in the previous 14 days and 38 (82%) were preceded by alerts within that time frame (*P*<.001; Figure 1). The sensitivity and specificity of alerts for predicting ED visits that occurred within 14 days following the alerts were 83% (95% CI 72-94) and 86% (95% CI 85-87), respectively. The positive and negative predictive values were 9.4% (95% CI 6.5-12.2) and 99.6% (95% CI 99.3-99.9), respectively, and the positive and negative likelihood ratios were 5.87 (95% CI 4.99-6.92) and 0.20 (95% CI 0.11-0.38), respectively (Table 4 and 5).

**Table 4:** Contingency table for alerts generated by the eHealth system following home health aide visits and for emergency department visits occurring within 14 days of the alerts, and accuracy assessment.

Characteristics	Home aides’ visits with subsequent alerts (n=2656)		*P* values
	Yes (n=405)	No (n=2251)	
Emergency department visits, n (%)	38 (18.5)	8 (0.3)	*<*.001
No emergency department visits, n (%)	367 (81.5)	2243 (99.7)	*<*.001

**Table 5:** Characteristics of alerts for predicting emergency department visits.

Home aides’ visits with subsequent alerts (n=2656)	Accuracy assessment (95% CI)
Sensitivity, % (95% CI)	83 (72-94)
Specificity, % (95% CI)	86 (85-87)
Positive likelihood ratio	5.87 (4.99-6.92)
Negative likelihood ratio	0.20 (0.11-0.38)
Positive predictive value, % (95% CI)	9.4 (6.5-12.2)
Negative predictive value, % (95% CI)	99.6 (99.3-99.9)

## Discussion

### Principal Results

In this intervention study, we successfully implemented an eHealth system based on HAs’ observations and a prediction algorithm that is capable of informing health care professionals of the risk of an ED visit in the next two weeks. In total, 109 HAs were involved in the study for 4753 visits. More than half of the visits were monitored. Alerts automatically displayed by this eHealth system accurately predicted emergency room visits, and 32% (131/405) of them were followed by interventions by the patients’ nurses or their general practitioners.

This eHealth system was well accepted and appreciated by HAs and their managers, and the accuracy was very good.

### Potential Bias and Levers

The evaluation of the accuracy of the alerts could have been biased by the transmission of the alert to a care manager. Nevertheless, it was shown that the interventions of the care managers allowed for a reduction in emergency hospitalizations, confirming the high predictive capacity of the system.

Balance between false negatives and false positives rates has been the subject of much reflection. In order to avoid a potential unnecessary ED visit (for a false positive, which is extremely rare), while avoiding as much as possible the loss of chances (false negative), the *F*_1_-score has been chosen for the best optimization between false positives and false negatives rates.

### Comparison With Prior Studies

The eHealth system reported in this study overcame the classic obstacles faced by such systems. First, the completion of the smartphone-based, customer-centered diagnostic tool was good, and 90% (72/81) of the HAs found it acceptable. This is in contrast to studies that have highlighted that barriers to the use of e-PROMs for caregivers or clinicians are primarily related to long completion time and poor usability [22]. Second, acceptance of the alerts was satisfactory, and the health professionals who received them produced a high response rate for health interventions. This is probably related to the reasonable number of alerts and the ratio of alerts per visit that did not overwhelm practitioners, achieved through the good specificity of the machine learning algorithm. These results contrast with those of other studies that report that practitioners, overburdened by automatic alerts, no longer contact patients to intensify treatment of symptoms despite appropriate daily monitoring [21,23,24]. Other features of the machine learning algorithm have contributed to the acceptance of this eHealth system; its supervised nature provided health care professionals with indications of changes in beneficiaries’ functional status (eg, ability to get up, move around, and eat, their mood, and loneliness) in addition to the alert alone; it helped them relieve their doubts and probably induced trust in the relevance of the alerts, since all the coordinating nurses found that information provided with alerts was clinically useful. It is likely that the acceptability rate would have been lower with deep learning algorithms that often have excellent predictive capabilities [25] because their operation is obscure to the users who receive the results, and that is a limitation when critical decisions need to be made.

Interestingly, in our study, the probability of an ED visit was very low after nurse or physician interventions following an alert, with a 10-fold decrease, compared to when alerts were not followed by such interventions. Even if this trial was not designed to examine such an outcome, this observation is very promising and prompts us to implement a controlled trial to document the effectiveness of the eHealth system. In addition, this device improves communication between professionals and promotes the empowerment of HAs. It responds to real public health issues for the prevention of the loss of autonomy in older people at home.

### Limitations

Our study faced some limitations. First, the study was conducted during the COVID-19 pandemic, which increased the risk of isolation for frail older adults and impacted primary care habits and HAs’ working conditions. In addition, the incidence of ED visits was lower compared to previous studies of participants with similar characteristics (13% vs 40%). This may be due to factors other than our intervention, such as reticence to attend EDs for fear of exposure to COVID-19 [26]. This also raises the question of lack of data on hospitalization or death causes, which could have allowed a more detailed analysis according to the context (eg, COVID-19). Investigations are in progress to understand death causes and the patient’s trajectory. Second, there is a limitation regarding deaths analysis; time of death was not taken into account, and it was not compared with the intervention’s date. Therefore, a causal link between the two cannot be made. Third, for the moment, the alert is displayed only when it is reliable (ie, with enough data); however, to avoid a potential loss of chance, the person’s condition and risks (geriatric and health) are transferred to the coordinating nurse, who can then assess the seriousness of the person’s situation (ie, informing the coordinating nurse that it will be necessary to have other questionnaires to display an alert). Finally, this trial was not controlled, and a trial with a randomized controlled design should be conducted to document its clinical efficacy and cost-effectiveness.

### Perspectives

This study opens up broad prospects for optimizing the relevance of emergency visits for frail older adults. The predictive algorithm based on longitudinal observations of HAs could be improved by other types of input, such as patient’s clinical or biological records or measurements from connected devices. In addition, our approach could be applied to new target events in older adults in specific health contexts, including oncogeriatrics, cardiogeriatrics, or postsurgery. Clinical investigations are currently in progress and will allow for investigating the transferability of this system to certain clinical contexts. Finally, the system might evolve to a decision support system to help health professionals to optimize and personalize ATHI.

### Conclusions

The eHealth system that we have successfully implemented offers an innovative approach to optimize the care of frail older adults. This approach is based on three paradigms: recording of the functional characteristics of daily life and their evolution over time, mobilization of nonprofessional health informants, and the use of a machine learning algorithm to monitor the level of individual risk and produce alerts that support health professional decisions for interventions. This means that multiple observers (not just social workers or nurses) could be trained to identify people at risk for ED visits. Such a predictive approach could form the basis for personalized health interventions that are designed to deliver early appropriate care and improve health outcomes.

## Appendix

Multimedia Appendix 1 The list of 23 items recorded by home care aides at each home visit and their completeness rates.

Multimedia Appendix 2 A simplified diagram of the processing system and the description of data processing system.

## Abbreviations

ATHI: alert-triggered health intervention
ED: emergency department
HA: home aide
PROM: patient-reported outcome measure

## Data Availability

The data that support the findings of this study are available from the corresponding author, JHV, upon reasonable request. Restrictions apply to the availability of the data collected by the home aids and the algorithm that was used in this study, which is the subject of a patent.
