# Supplementary material for: Real-world Implementation of an eHealth System Based on Artificial Intelligence Designed to Predict and Reduce Emergency Department Visits by Older Adults: Pragmatic Trial
Source: J Med Internet Res. 2022 Sep 8;24(9):e40387. doi: 10.2196/40387 (PMC9501682; doi:10.2196/40387)
Supplement: Multimedia Appendix 1 [file jmir_v24i9e40387_app1.docx]

*List of 23 items recorded by the home care aides at each home visit and their completeness rates.*

| Items related to | Items | Completeness rate (%) |
| --- | --- | --- |
| Activities of daily living | The person has groomed him/herself | 100 |
|  | The person gets out of bed | 100 |
|  | The person is able to move in his/her home | 100 |
|  | The person has moved out of the home | 100 |
|  | The person has prepared his/her meal | 100 |
|  | The person has eaten | 100 |
| Possible medical symptoms | The person seems tired | 100 |
|  | The person seems feverish | 100 |
|  | The person is painful | 100 |
|  | The person has trouble breathing | 100 |
|  | The person has swollen legs | 100 |
|  | The person has fallen | 100 |
|  | The person seems better than at the last visit | 100 |
| Behavioral changes | The person places objects in inappropriate places | 100 |
|  | The person is aggressive | 100 |
|  | The person does not recognize me | 100 |
|  | The person has forgotten when I came | 100 |
|  | The person has refused help for grooming | 100 |
|  | The person communicates inconsistently | 100 |
| Communication changes | The person communicates little | 100 |
|  | The person seems sad | 100 |
|  | The person seems indifferent | 100 |
|  | The person has no visit from, or contact with his/her social support | 100 |
